# Dual antigen-sampling sites support early mucosal antigen uptake in the porcine nasal cavity

**DOI:** 10.1186/s13567-026-01863-2

**Published:** 2026-10-27

**Authors:** Shubin Li, Yuye Liu, Minjie Wang, Haonan Li, Kang Chen, Yifei Zhu, Yulu Bao, Qiang Zhang, Qian Yang

**Affiliations:** 1 MOE Joint International Research Laboratory of Animal Health and Food Safety, College of Veterinary Medicine, Nanjing Agricultural University, Nanjing, Jiangsu China; 2 Novo Biotech Co., Ltd., Shaoxing, 312366 China

**Keywords:** Porcine nasal cavity, nasopharynx-associated lymphoid tissue, nasal M-like cells, antigen sampling, intranasal vaccination

## Abstract

Intranasal vaccination is a promising strategy for preventing respiratory infections. However, the anatomical and cellular pathways responsible for antigen sampling in the porcine nasal cavity remain incompletely understood. Here, we identified and characterized two spatially distinct antigen-sampling sites in the porcine nasal cavity: the anterior respiratory mucosa (RM) and the posterior nasopharynx-associated lymphoid tissue (NALT). Following intranasal administration of ovalbumin (OVA), a subset of OVA^+^ epithelial cells at both sites was labeled by *Ulex europaeus* agglutinin I (UEA-1) and NKM 16-2-4, indicating that these cells might exhibit an M-cell-like phenotype. In RM, OVA was initially localized to the epithelium and was subsequently detected in the lamina propria by 6 h and in CD11c^+^ cells (a population that might include dendritic cells) at 24 h. By contrast, OVA reached the underlying lymphoid follicles in NALT as early as 2 h. OVA^+^ cells were also detected in mandibular draining lymph nodes at 6 h. Moreover, an air–liquid interface co-culture model showed apical-to-basolateral OVA transport across differentiated nasal epithelial cultures and subsequent OVA acquisition by underlying differentiated bone marrow cells (dBMCs). Together, these findings identify anterior RM and posterior NALT as two antigen-sampling sites with distinct temporal dynamics and define early cellular events that might contribute to mucosal immune induction in pigs.

**Supplementary information:**

The online version contains supplementary material available at https://doi.org/10.1186/s13567-026-01863-2.

## Introduction

The porcine nasal mucosa serves as a major portal of entry for numerous viral and bacterial pathogens. These include porcine reproductive and respiratory syndrome virus, swine influenza virus, pseudorabies virus, *Mycoplasma hyopneumoniae*, *Bordetella bronchiseptica*, and *Pasteurella multocida*, which collectively cause substantial economic losses to the swine industry [1–4]. Intranasal immunization could induce local mucosal immune responses at the initial site of pathogen exposure, thereby limiting pathogen entry and infection [5–7]. Several intranasal vaccination strategies have therefore been evaluated in pigs. For instance, Dwivedi et al. demonstrated that intranasal delivery of an attenuated porcine reproductive and respiratory syndrome virus (PRRSV) vaccine with a *Mycobacterium* lysate adjuvant enhanced protection against challenge infection in pigs [8]. Similarly, intranasal formulations of inactivated swine influenza virus combined with chitosan or innate immune stimulators enhanced mucosal immune responses in porcine models [9–11]. However, the cellular and anatomical mechanisms underlying immune induction in the porcine nasal cavity remain poorly understood.

Mucosal immune inductive sites are specialized anatomical regions in which luminal antigens are sampled and antigen-specific immune responses are initiated [12]. A hallmark of these sites is the presence of antigen-sampling microfold (M) cells, which are specialized to sample luminal antigens and transport them to underlying immune cells [13]. Nasopharynx-associated lymphoid tissue (NALT), located in the posterior nasal cavity, is an established inductive site for nasal antigens [14]. M cells within the NALT follicle-associated epithelium (FAE) mediate antigen uptake and transcytosis, thereby linking luminal antigen sampling to the activation of the underlying immune compartment [15, 16]. Consistent with this function, recent studies have shown that NALT contributes to the generation of immunoglobulin A (IgA)-secreting cells after intranasal vaccination [14]. Nevertheless, nasal IgA responses can also develop in the absence of NALT, suggesting that additional anatomical sites may contribute to nasal immune induction [17]. In mice, a distinct population of M cells has been identified in the anterior nasal respiratory epithelium. These cells exhibit strong antigen-sampling activity and may provide an alternative portal for mucosal antigen uptake outside the posterior NALT [18]. These findings raise the possibility that the anterior respiratory mucosa and posterior NALT represent spatially distinct antigen-sampling compartments within the nasal cavity. However, whether comparable functional M-like cells and dual antigen-sampling sites exist in pigs remains unknown. Defining the distribution and function of porcine nasal M-like cells is therefore important for understanding the early events of nasal immune induction and optimizing intranasal vaccine delivery in swine.

Antigen acquisition by antigen-presenting cells (APCs) is a critical early event in responses to nasal vaccination [19]. In rodent models, APCs of NALT are positioned beneath or near M cells, facilitating the transfer of luminal antigens to the underlying immune compartment [20]. However, such a mechanism has not been investigated in swine. Although abundant APCs have been identified in the respiratory mucosa of the porcine nasal cavity [21], whether these cells acquire luminal antigens through putative M-like epithelial cells remains unclear.

In this study, ovalbumin (OVA), a well-characterized and readily traceable model protein antigen, was used to investigate antigen sampling in the porcine nasal cavity [22, 23]. We compared the anterior respiratory mucosa and posterior NALT, characterized OVA-sampling epithelial cells with M-cell-like features, and assessed the spatiotemporal transfer of OVA to underlying immune-cell populations. Using a co-culture model of primary nasal epithelial cells and interleukin (IL)−4/granulocyte-macrophage colony-stimulating factor (GM-CSF) differentiated bone marrow cells (dBMCs), we further examined transepithelial antigen transport and subsequent antigen acquisition by dBMCs. These analyses define complementary anterior and posterior pathways of early nasal antigen sampling in pigs.

## Materials and methods

### Animals

Conventional Duroc × (Landrace × Yorkshire) hybrid piglets were obtained from a commercial farm in Nanjing, China. Detailed information on the pigs used in this study—including age, sample size, treatment, sampling time, tissues collected, experimental purpose, and associated analyses—is summarized in Additional file 1. Piglets were euthanized by intravenous administration of sodium pentobarbital (100 mg/kg) before tissue collection.

### OVA uptake by porcine nasal epithelia

Purified OVA (Yeasen, China) was diluted in phosphate-buffered saline (PBS) and used in this study. To assess antigen-sampling cells, 7-day-old piglets (*n* = 3 per group) received intranasal administration of OVA (500 μL into each nostril, 1 mg/mL) or an equal volume of PBS. At 2 h after administration, piglets were euthanized and RM and NALT were collected, fixed in 4% paraformaldehyde, and paraffin-embedded as described previously [24]. Immunohistochemistry (IHC) and immunofluorescent assays were performed on nasal tissue to detect OVA^+^ cells. For each nasal region (I–V), OVA^+^ cells were counted in three nonconsecutive sections separated by more than 200 μm from each piglet. The counts from the three sections were averaged to generate one value for each region in each piglet. Values from three piglets were then used as independent biological replicates for statistical analysis (*n* = 3).

### Immunohistochemistry

Paraffin sections were deparaffinized in xylene and rehydrated through a graded ethanol series.

Heat-induced antigen retrieval was performed in citrate buffer (pH 6.0) at 95–100 °C for 20 min. Sections were blocked with 5% bovine serum albumin (BSA) and incubated overnight at 4 °C with rabbit anti-OVA antibody (1:200; Novus Biologicals, USA). Signals were developed using a streptavidin–biotin complex kit (Boster Bio, China) and 3,3′-diaminobenzidine substrate according to the manufacturers’ instructions. Brown cytoplasmic staining was considered OVA^+^. Sections processed without primary antibody served as negative controls. Detailed information on antibodies used in this study are listed in Additional file 2.

### Transmission and scanning electron microscopy

Nasal mucosal tissues from the RM and NALT of one healthy 7-day-old piglet were collected for transmission electron microscopy (TEM) and scanning electron microscopy (SEM). Samples were fixed in 2.5% glutaraldehyde at 4 °C for 24 h. For TEM, the tissues were cut into approximately 1 mm^3^ pieces, dehydrated through a graded ethanol series, infiltrated with epoxy resin, and embedded for polymerization. Ultrathin sections (70–90 nm) were prepared using an ultramicrotome, collected on copper grids, stained with uranyl acetate and lead citrate, and examined using a transmission electron microscope (H-7650, Hitachi, Japan). For SEM, the tissues were trimmed into approximately 5 mm × 5 mm pieces, washed with phosphate buffer, and postfixed with 1% osmium tetroxide for 1–2 h. After dehydration through a graded ethanol series, the samples were critical-point dried and mounted on aluminum stubs with the epithelial surface facing upward. The samples were then coated with an approximately 10-nm-thick gold layer using an ion sputter coater. The epithelial surface morphology was examined using a scanning electron microscope (SU8010; Hitachi, Japan).

### Immunofluorescent assay to identify the features of OVA-sampling epithelial cells

To identify the cell types involved in protein antigen sampling, RM and NALT tissue sections were deparaffinized, rehydrated, and subjected to antigen retrieval as described for immunohistochemistry. After blocking with 5% BSA, the sections were incubated overnight at 4 °C with rabbit anti-OVA antibody (Novus Biologicals, USA) together with the indicated cell marker antibodies, including mouse anti-KRT5, mouse anti-α-tubulin, mouse anti-MUC5AC, mouse anti-OMP, and rat NKM 16-2-4 antibodies. The sections were then incubated for 1 h at 37 °C with the appropriate secondary antibodies: YSFluor^®^ 594-conjugated goat anti-rabbit IgG, YSFluor^®^ 488-conjugated donkey anti-mouse IgG, or YSFluor^®^ 647-conjugated donkey anti-rat IgG (Yeasen, China). For lectin staining, sections were processed through the blocking step as described above and incubated overnight at 4 °C with fluorescein isothiocyanate (FITC)-conjugated UEA-1 (Sigma-Aldrich, Germany). Nuclei were counterstained with 4′,6-diamidino-2-phenylindole (DAPI), and fluorescence images were acquired using a confocal microscope (LSM 900; Zeiss, Germany). Detailed information on the antibodies and reagents used in this study is provided in Additional file 2.

### Spatiotemporal tracking of OVA after intranasal administration

To track the distribution of the protein antigen after intranasal delivery, 7-day-old piglets (*n* = 3/time point) were euthanized at 2, 4, 6, and 24 h after intranasal OVA administration. RM, NALT, and draining lymph node (DLN) tissue were fixed in 4% paraformaldehyde, decalcified in 10% ethylenediaminetetraacetic acid (EDTA)—RM tissue only, paraffin-embedded, and sectioned at 5 μm as described previously. OVA^+^ cells were identified by immunohistochemistry (IHC). Tissues from PBS-treated piglets were used as a negative control.

To identify the phenotypic characteristics of OVA^+^ cells in the lamina propria of RM, multiplex immunofluorescence staining was performed using a tyramide signal amplification (TSA) kit (Aifang Biotechnology, China). Sections were incubated with the anti-OVA antibody overnight at 4 °C, followed by a horseradish peroxidase (HRP)-conjugated secondary antibody and TSA 594. After heat-mediated antibody stripping, the sections were incubated with the anti-CD11c antibody overnight at 4 °C, followed by an HRP-conjugated secondary antibody and TSA 488. Following a second round of heat-mediated antibody stripping, the sections were incubated with NKM 16-2-4 overnight at 4 °C and subsequently with YSFluor^®^ 647-conjugated donkey anti-rat IgG for 30 min at room temperature. After completion of all staining procedures, nuclei were counterstained with DAPI. Images were acquired using a confocal microscope (LSM 900; Zeiss, Germany).

### Isolation and differentiation of porcine bone marrow cells

Bone marrow cells were isolated from the femurs of three 30-day-old piglets and cultured in the presence of GM-CSF and IL-4 for 7 days to generate differentiated bone marrow cells (dBMCs) [21]. After erythrocyte lysis, cells were cultured in Roswell Park Memorial Institute (RPMI) 1640 medium supplemented with 10% fetal bovine serum (FBS), 1% penicillin–streptomycin, 20 ng/mL GM-CSF, and 10 ng/mL IL-4. At 60 h, the medium was replaced to remove nonadherent granulocytes. On day 6, cells were harvested and replated overnight to reduce adherent-cell contamination. dBMCs were collected on day 7 for experiments or cryopreservation.

### Isolation and culture of the primary porcine nasal epithelial cells

The heads of three 30-day-old pigs were longitudinally bisected along the nasal septum to expose the nasal cavity. The nasal septum was carefully reflected using sterile scissors, and the nasal turbinates were excised. The nasal septum and turbinate tissues were collected and immediately immersed in 500 mL of Hank’s solution supplemented with antibiotics (Fungizone, 2.5 μg/mL; vancomycin, 100 μg/mL; and colomycin, 16 μg/mL). The nasal mucosa was stripped from the underlying cartilage and washed with cold Hank’s solution containing antibiotics. After being minced into 1–2 mm^3^ pieces, the tissues were digested overnight at 4 °C in Hank’s solution supplemented with collagenase IV (10 mg/mL; Yeasen, China) and DNase I (100 U/mL, Sigma, Germany). The digested tissues were centrifuged at 125 × *g*, washed with PBS, and further digested with 0.25% trypsin for 10 min at 37 °C. Digestion was then terminated by adding Hank’s solution containing 10% FBS, and the cell suspension was filtered through a 100-μm cell strainer. After centrifugation, the cell pellet was collected and resuspended in expansion medium (PneumaCult™-NGEx; STEMCELL Technologies, Canada) supplemented with Y-27632 (10 μM; Selleck, USA). The cells were seeded into tissue-culture-treated T25 flasks and cultured for 3–6 days until they reached 60–80% confluence. The cells were then passaged for further expansion or cryopreserved. The nasal epithelial cells were trypsinized and seeded at a density of 40 000 cells per well onto collagen IV-coated Transwell inserts (3470, 6.5 mm; 0.4-μm pore size; Corning, USA). The lower and upper chambers were filled with 0.6 and 0.2 mL of medium, respectively. Once a confluent monolayer had formed after 3–6 days, the apical medium was removed to establish an air–liquid interface (ALI), and the medium in the basolateral chamber was replaced with differentiation medium (PneumaCult™-ALI; STEMCELL Technologies, Canada). The differentiation medium was refreshed every 2 days. Beginning 2 weeks after airlift, excess mucus on the apical surface was removed by washing with Dulbecco’s PBS (D-PBS) once weekly. Fully differentiated ALI-cultured nasal epithelial cells (ALI-NECs), obtained after more than 21 days of differentiation, were used in the subsequent experiments.

### Identification and functional assessment of nasal M-like cells in ALI-NECs

Immunofluorescence staining was performed to identify nasal M-like cells in ALI-NECs. The apical surface of the ALI-NECs was washed three times with Hank’s solution, followed by the addition of 200 μL OVA solution (500 μg/mL) to the apical chamber. A swine tracheal epithelial cell line (STEC), kindly provided by Professor Hongjie Fan (College of Veterinary Medicine, Nanjing Agricultural University), was used as a control [25]. After 2 h of incubation, the cells were washed with Hank’s solution, fixed with 4% paraformaldehyde, and stained with NKM 16-2-4 and anti-OVA antibodies. Nuclei were counterstained with DAPI, and fluorescence images were acquired using a confocal microscope (LSM 900; Zeiss). Fluorescence images acquired at 20× magnification were analyzed using ImageJ. OVA^+^ cells were classified as NKM 16-2-4 positive or negative using consistent thresholding criteria, and their proportions were calculated relative to the total number of OVA^+^ cells. One field was analyzed for each independent biological replicate (*n* = 3; Figure 5C).

To assess transepithelial OVA transport, separate Transwell inserts were assigned to each time point. Undiluted basolateral medium was collected once from each insert at 0, 2, 6, or 12 h after apical OVA addition and analyzed using an OVA enzyme-linked immunosorbent assay (ELISA) kit (Cloud–Clone, China) according to the manufacturer’s instructions. To assess the contribution of membrane fluidity-dependent vesicular transport, ALI-NECs and STEC monolayers were preincubated at 4 °C before apical OVA exposure and maintained at 4 °C throughout the transport assay. Parallel inserts that were maintained at 37 °C served as controls. Basolateral OVA concentrations were subsequently measured by ELISA at 2 h post-administration [26].

### Establishment of the NECs–dBMCs co-culture system

NECs–dBMCs co-culture: Transwell inserts containing NECs were inverted in a 12-well plate after airlift completion, and 5 × 10^5^ carboxyfluorescein succinimidyl ester (CFSE)-labeled dBMCs (labeling with CFSE) were seeded on the basolateral side for 4 h. Then, the Transwell inserts were returned to the upright position and replaced in 24-well plates with medium in the lower chamber for further procedures. OVA (500 μg/mL) was added to the apical chamber for at least 2 h. After removal of OVA and washing, the cells were maintained under air–liquid interface conditions for an additional 6 h. Then, immunofluorescence analysis was performed to detect the OVA uptake by dBMCs.

### Fucosylation inhibition assay

ALI-NECs were treated with the fucosylation inhibitor 2F-Fuc at 0, 50, or 100 μM for 3 days before apical exposure to OVA (200 μL, 500 μg/mL) for 2 h. The cells were subsequently fixed and stained with NKM 16-2-4 to detect α-(1,2)-fucosylation and an anti-OVA antibody to detect internalized OVA. Colocalization between α-(1,2)-fucosylation and OVA was examined by confocal microscopy. Immunofluorescence images acquired at 20× magnification were analyzed using ImageJ software (NIH, USA). One field was analyzed for each independent biological replicate (*n* = 3). DAPI^+^ nuclei were counted to determine the total number of cells, and NKM 16-2-4^+^ and OVA^+^ cells were independently quantified using consistent thresholding criteria. The percentages of NKM 16-2-4^+^ and OVA^+^ cells were calculated relative to the total number of DAPI^+^ cells (Figure 5H).

In the NEC–dBMC co-culture model, ALI-NECs pretreated with 2F-Fuc or vehicle control were co-cultured with differentiated bone marrow cells (dBMCs). OVA–APCs were added to the apical chamber at 2 μg/mL and incubated for 2 h. The apical medium was then removed, and the cells were cultured for an additional 6 h under air–liquid interface conditions. NECs and dBMCs were then separately harvested by trypsinization. NECs were stained with UEA-1–FITC, whereas dBMCs were stained with SWC3a-PE and SLA-DR–FITC, followed by flow cytometric analysis of OVA uptake.

### Measurement of TEER

Transepithelial electrical resistance (TEER) was assessed using a Millicell Electrical Resistance System (ERS) volt-ohmmeter (Millipore, Molsheim, France). Only inserts with baseline TEER values of at least 500 Ω·cm^2^ were used for subsequent experiments. The apical medium was removed to reestablish the air–liquid interface (ALI). After OVA addition, TEER was measured at 0, 2, 6, and 12 h, and medium-treated inserts served as controls. The resistance of an empty insert was recorded as a blank control, and its value was subtracted from that of each sample. Computational formula: TEER (Ω·cm^2^) = [R_sample_ (Ω) − R_blank_ (Ω)] × 0.33 cm^2^.

### Single‑cell RNA-sequencing

The single-cell RNA-sequencing (scRNA-seq) data of healthy 7 day-old piglets’ nasal mucosa are available in the gene expression omnibus (GEO) database with accession number GSE274334 [27]. Single-cell capture was performed using a Chromium Controller instrument (10× Genomics, CA, USA) as previously described [27]. The mucosa of the nasal respiratory region was dissected from four 7-day-old piglets (two porcine nasal mucosae were mixed into one sample). The tissues were digested and resuspended as single suspensions. The viability of the cell suspension was determined by trypan blue staining, and the live cell concentration was adjusted to 1000–2000 cells/µL. The cells were then captured with a Chromium Single Cell Instrument (10× Genomics) after binding to barcoded gel beads. The raw scRNA-seq data were obtained after reverse transcription and RNA sequencing. These processes were completed by Gene Denovo (Guangzhou, China).

### Single-cell RNA-sequencing data processing

The raw sequencing data were processed using Cell Ranger software (version 3.1.0, 10 × Genomics, USA), and the sequence reads were aligned to the porcine reference genome (Sscrofa 11.1). Single-cell analysis was performed using Seurat version 5.3.0. After quality control, LogNormalize, variable gene selection, principal component analysis (PCA), and Harmony integration, cell clustering (resolution: 1.0) and *t*-distributed stochastic neighbor embedding (*t*-SNE) were run. Finally, we loaded the log-normalized matrices on the SingleR package for cell type annotation. Further manual annotation was performed based on reported specific marker genes. Marker genes used in this study were listed in Additional file 3.

### Flow cytometric analysis

Nasal swab-derived cells: 7-day-old piglets were intranasally administered PBS or OVA (500 μL per nostril, 1 mg/mL), and nasal swab-derived cells were collected 2 h later (*n* = 3 per group). Swabs were agitated in sterile Hank’s balanced salt solution (HBSS) for 15 s, and released cells were collected by centrifugation at 250 × *g* for 5 min at 4 °C. After washing, cells were digested with collagenase IV (10 mg/mL; Yeasen, China) and deoxyribonuclease I (DNase I; 100 U/mL; Sigma-Aldrich, Germany) at 37 °C for 30 min, followed by erythrocyte lysis. Cells were adjusted to 2 × 10^6^ per sample and stained with Zombie NIR Fixable Viability Dye (BioLegend, USA) according to the manufacturer’s instructions, followed by UEA-1–FITC staining for 30 min at 4 °C. Cells were then fixed and permeabilized with BD Cytofix/Cytoperm™ solution (BD Biosciences, USA) for 20 min at 4 °C, followed by two washes with BD Perm/Wash™ buffer. For intracellular OVA detection, permeabilized cells were sequentially incubated with rabbit anti-OVA antibody (Abcam, UK) and APC-conjugated donkey anti-rabbit IgG in perm/wash buffer. After washing and filtration through a 40-μm cell strainer, samples were analyzed using a CytoFLEX flow cytometer (Beckman Coulter, USA). Dead cells were excluded based on Zombie NIR staining.

RM- and NALT-derived cells: to evaluate antigen uptake by nasal mucosal antigen-presenting cells, respiratory mucosa (RM) and nasopharynx-associated lymphoid tissue (NALT) were collected from OVA-treated piglets at 6 h and 24 h post-administration. The tissues were minced into approximately 1-mm pieces and washed five times with cold HBSS to remove mucus. Tissue fragments were collected by centrifugation at 250 × *g* for 5 min at 4 °C and digested in Hank’s solution containing collagenase IV (10 mg/mL; Yeasen, China) and DNase I (Sigma-Aldrich, Germany) at 37 °C for 30 min. The digested tissues were mechanically dissociated through a 100-μm stainless-steel cell strainer. Cells were collected by centrifugation, and erythrocytes were removed by hypotonic lysis. The resulting cells were counted, adjusted to 2 × 10^6^ cells per sample, and stained with FITC-conjugated anti-porcine SLA-DR and PE-conjugated anti-porcine SWC3a antibodies for 30 min at 4 °C. After washing, the cells were sequentially incubated with rabbit anti-OVA antibody (Abcam, UK) and APC-conjugated donkey anti-rabbit IgG (Yeasen, China). The samples were filtered through a 40-μm cell strainer, stained with 7-aminoactinomycin D (7-AAD) immediately before acquisition, and analyzed using a BD FACSCalibur flow cytometer (BD Biosciences, USA).

ALI-NECs: OVA–FITC (2 μg/mL; Bioss, China) was added to the apical chamber of ALI-NECs for 2 h. The cells were subsequently washed with PBS, dissociated with 0.25% trypsin (BioChannel, China), and stained with PE-conjugated NKM 16-2-4 (4 °C, 30 min) and 7-AAD before flow cytometric analysis.

NEC–dBMCs co-culture: OVA–APC (2 μg/mL) was added to the apical chamber for at least 2 h. After removal of OVA and washing, the cells were maintained under air–liquid interface conditions for an additional 6 h. Then, NECs and dBMCs were collected separately. NECs were stained with UEA-1-FITC, whereas dBMCs were stained with SLA-DR–FITC and SWC3a–PE (4 °C, 30 min) before flow cytometric analysis. 7-AAD was used to exclude the dead cells.

For all analyses, Zombie NIR or 7-AAD^+^ dead cells were excluded, and negative cells were defined as viable cells. Data acquired using the CytoFLEX flow cytometer were collected with CytExpert software (Beckman Coulter, USA), and all flow cytometric data were subsequently analyzed using FlowJo software (BD Biosciences, USA). The gating strategies are shown in Additional file 6.

### Statistical analysis

Statistical analyses were performed using GraphPad Prism 10 (GraphPad Software, USA). Data are presented as mean ± standard deviation (SD). Individual piglets or independent pig-derived cell cultures were considered biological replicates, as specified in the corresponding figure legends. For regional quantification in tissue sections, measurements from multiple sections of the same piglet were averaged to obtain a single value for that animal before statistical analysis. Comparisons between two independent groups were performed using two-tailed unpaired *t* tests, whereas paired measurements obtained from the same biological replicate were analyzed using two-tailed paired *t* tests. Comparisons among three or more independent groups were performed using one-way analysis of variance (ANOVA) followed by Tukey’s or Dunnett’s multiple-comparisons test, as appropriate. When multiple conditions or anatomical regions were evaluated within the same biological replicate, a one-way repeated-measures ANOVA followed by the indicated multiple-comparisons test was used. Experiments involving two factors with repeated measurements were analyzed using two-way repeated-measures ANOVA followed by Šídák’s multiple-comparisons test. For all parametric analyses, the assumptions of approximate normality and homogeneity of variance were evaluated before statistical testing. For repeated-measures analyses, the within-subject structure of the data was accounted for, and the assumption of sphericity was evaluated where applicable. Appropriate corrections were applied when the sphericity assumption was violated. When the assumptions required for parametric testing were not adequately satisfied, the corresponding nonparametric test was used. The specific statistical test and multiple-comparisons procedure used for each experiment are indicated in the corresponding figure legend. The age-series experiment was analyzed qualitatively and was not subjected to statistical comparison. A *P* value < 0.05 was considered statistically significant.

## Results

### OVA-sampling epithelial cells occur in anterior RM and posterior NALT

**Figure 1:**
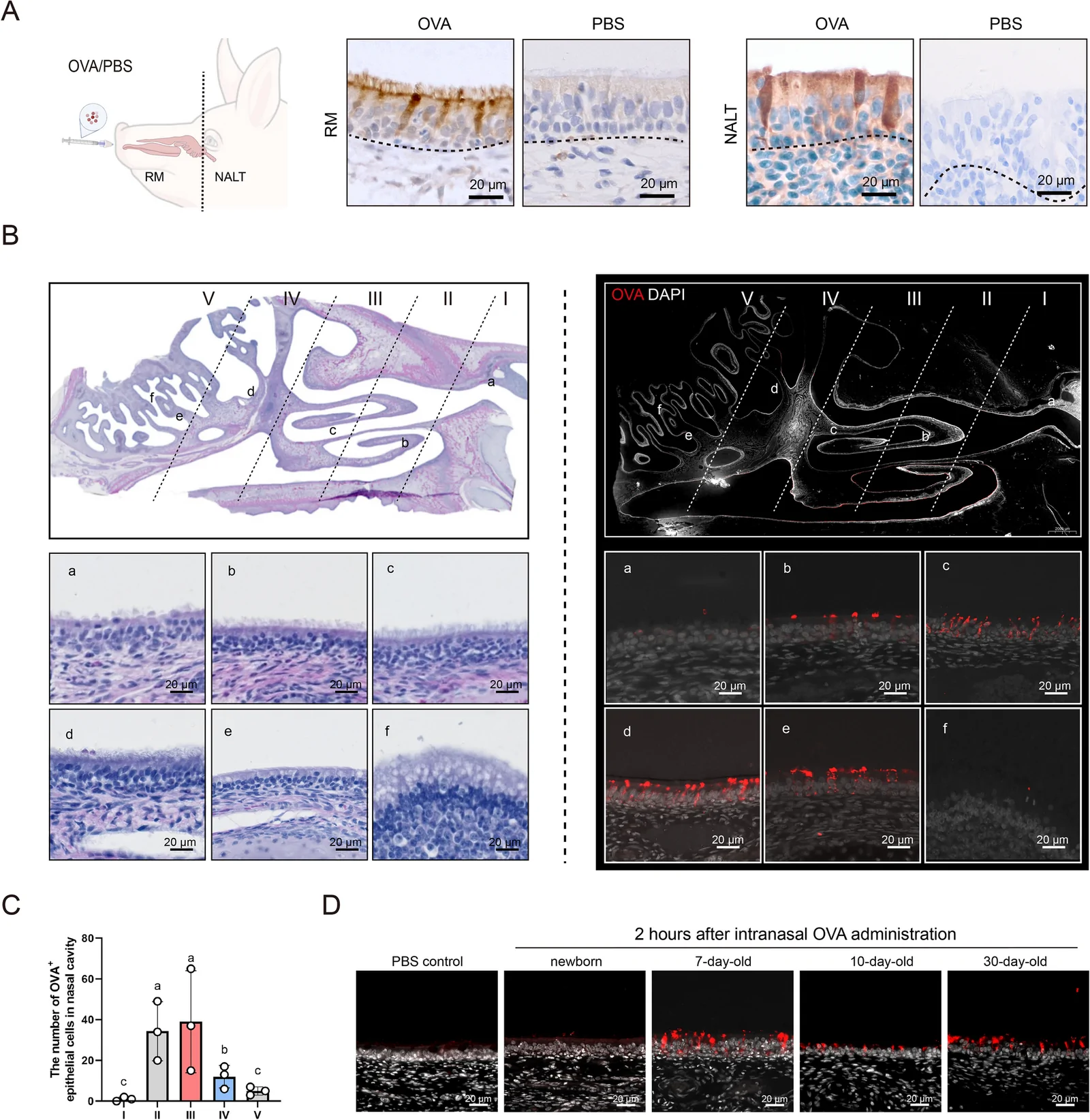
OVA-sampling epithelial cells in porcine RM and NALT. A 7-day-old piglets received intranasal ovalbumin (OVA) or PBS, and nasal tissues were collected 2 h later. The schematic indicates the anterior respiratory mucosa (RM) and posterior nasopharynx-associated lymphoid tissue (NALT). OVA was detected by immunohistochemistry; PBS-treated tissues served as negative controls. Dashed lines separate the epithelium (upper) from lamina propria (lower) in RM or underlying lymphoid follicle in NALT (lower). **B** Hematoxylin and eosin staining of a longitudinal nasal section and immunofluorescence staining (targeting OVA) in an adjacent section. The nasal cavity was divided into regions I–V as described previously [27]. Panels a–f show representative areas. **C** Quantification of OVA^+^ epithelial cells across regions I–V. Data are presented as mean ± SD; *n* = 3 piglets. Different letters indicate significant differences between groups (one-way repeated-measures ANOVA followed by Tukey’s multiple-comparisons test, *P* < 0.05), whereas groups sharing at least one letter are not significantly different. **D** Immunofluorescent assay targeting OVA was performed in nasal tissues of region III from newborn and 7-, 10-, and 30-day-old piglets. PBS-treated tissue served as a negative control.

Piglets (7 days old) were used for the initial characterization of nasal antigen sampling because young piglets are a clinically relevant target population for intranasal immunization. At 2 h after intranasal OVA administration, OVA^+^ epithelial cells were detected in both the anterior respiratory mucosa (RM) and the follicle-associated epithelium (FAE; above the dashed line) overlying posterior NALT (Figure 1A). Based on our previously established anatomical and histological classification, the porcine nasal cavity was divided into five regions: region I corresponds to the nasal vestibule, regions II–IV comprise the respiratory region, and region V represents the olfactory region [24]. In longitudinal nasal sections, OVA^+^ cells were distributed mainly throughout regions II-IV, with the highest numbers in regions II and III (Figure 1B, C). No OVA^+^ cells were detected in the nasal vestibule (region I). In region V, OVA^+^ cells were restricted to the respiratory–olfactory transition zone and were not detected in mature olfactory epithelium (Figure 1B). In region III, OVA-sampling epithelial cells were not observed in the examined sections from newborn piglets but were present in 7-, 10-, and 30-day-old piglets (Figure 1D).

### OVA-sampling epithelial cells display M-cell-like features

**Figure 2:**
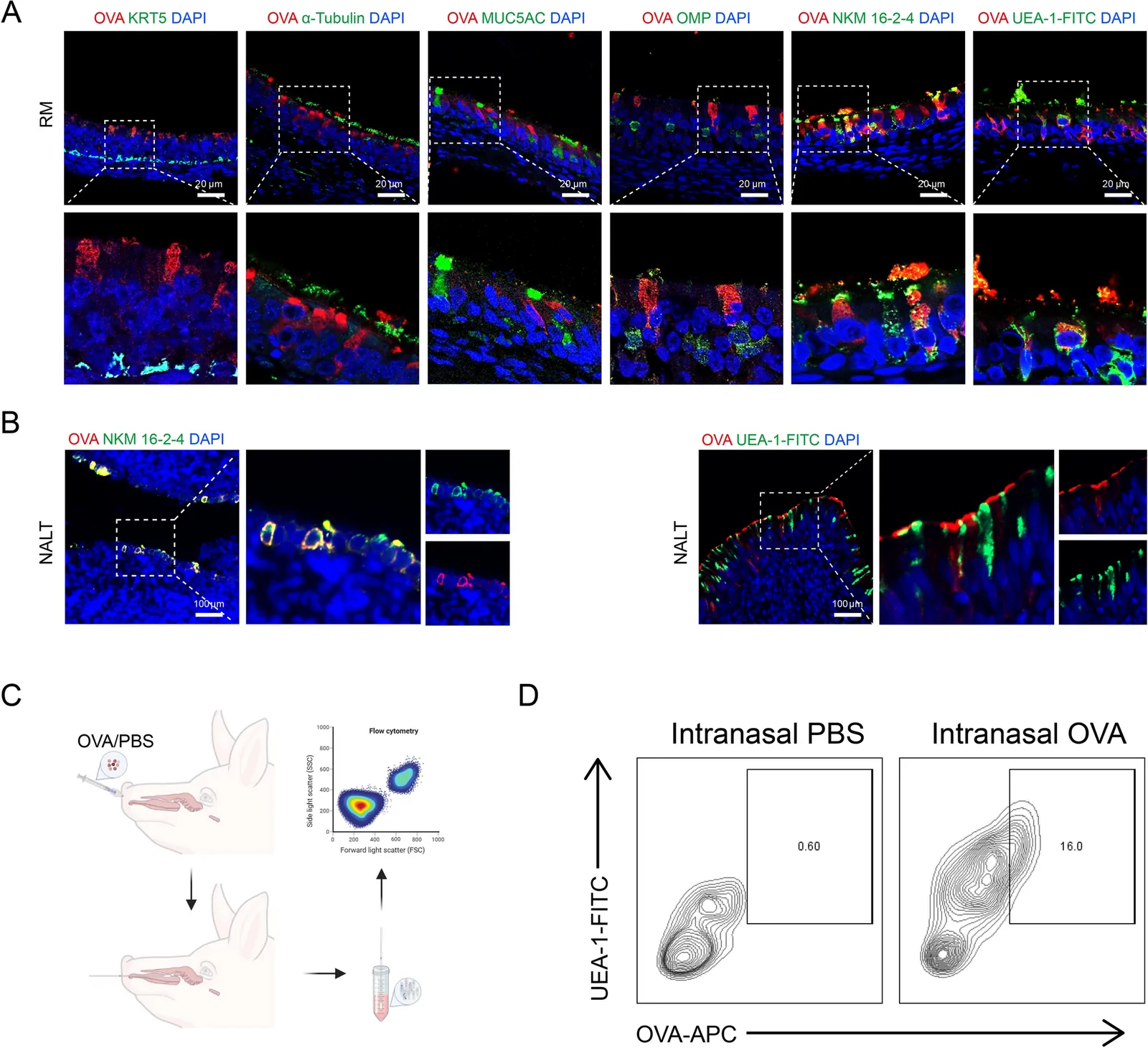
OVA-sampling epithelial cells display M-cell-like features. A Representative immunofluorescence images of respiratory mucosa (RM) stained for ovalbumin (OVA) and KRT5, α-tubulin, MUC5AC, OMP, NKM 16-2-4, or UEA-1-FITC. Enlarged views of boxed regions are shown below. **B** Representative nasopharynx-associated lymphoid tissue (NALT) sections showing OVA with NKM 16-2-4 or UEA-1. Nuclei were counterstained with DAPI. **C** Schematic of nasal swab collection 2 h after intranasal OVA or PBS administration. **D** Representative flow cytometry plots of UEA-1-FITC and OVA staining in viable nasal swab-derived cells. Values in the upper-right quadrant indicate the percentage of UEA-1^+^OVA^+^ cells within the parent population.

To characterize OVA-sampling epithelial cells, we examined their association with epithelial cell markers. No convincing intracellular colocalization was observed between OVA and KRT5, MUC5AC, OMP, or α-tubulin. UEA-1 and NKM 16-2-4, both of which recognize α-(1, 2)-fucose-containing glycans associated with mouse M cells [18, 28], labeled many OVA-sampling cells in RM and NALT (Figure 2A, B). Flow cytometric analysis of nasal swab-derived cells further showed that a subset of OVA^+^ nasal swab-derived cells was UEA-1^+^ (Figure 2C, D). Together with their antigen-sampling phenotype, these findings support the presence of porcine nasal epithelial cells with M-cell-like features. α-(1,2)-Fucosylated cells were also detected in nasal and tracheal epithelia and across multiple age groups (Additional file 4 A, B).

To determine whether a transcriptionally distinct M-cell-like populations could be resolved, we reanalyzed our previously published porcine nasal scRNA-seq dataset [27]. A total of 17 annotated cell populations were identified, including a club-cell lineage that can give rise to airway M cells in humans (Additional file 5A, B) [27, 29–31]. Among reported respiratory M-cell-associated genes, *MARCKSL1* and *ANXA5* were broadly detectable, whereas the more canonical markers *SOX8*, *SPIB*, *CCL9*, *CCL20*, *GP2*, *TNFRSF11A*, and *TNFAIP2* did not define a discrete cluster (Additional file 5C). Thus, no transcriptionally distinct M-cell cluster was resolved in this dataset.

### Histological and ultrastructural features of RM and NALT

**Figure 3:**
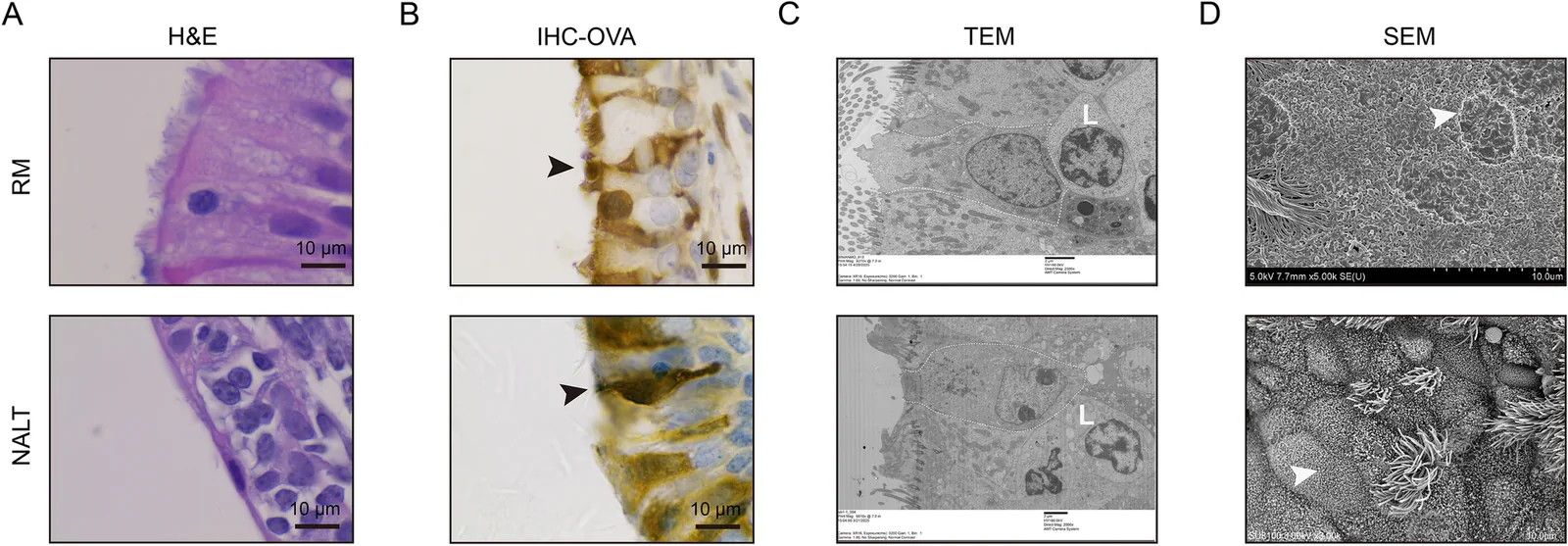
Histological and ultrastructural features of anterior RM and posterior NALT. A Representative hematoxylin and eosin staining images of the epithelium in anterior respiratory mucosa (RM) and posterior nasopharynx-associated lymphoid tissue (NALT). **B** Ovalbumin (OVA) immunohistochemistry in RM and NALT; arrowheads indicate OVA^+^ epithelial cells. **C** Representative transmission electron micrographs from a 7-day-old piglet. Dotted outlines indicate putative M-like epithelial cells, and “L” indicates an adjacent lymphoid cell. **D** Representative scanning electron micrographs of RM and NALT surfaces; arrowheads indicate cells with short, irregular apical microvilli or protrusions.

Histological analysis revealed distinct epithelial architectures at the two antigen-sampling sites. Anterior RM was lined by pseudostratified ciliated columnar epithelium, whereas the epithelium overlying posterior NALT was thinner and predominantly nonciliated (Figure 3A). OVA^+^ cells at both sites exhibited a columnar morphology with apical microvilli (Figure 3B). The ultrastructural features of the two antigen-sampling sites were further compared by transmission electron microscopy. In samples from a healthy 7-day-old piglet, epithelial cells with irregular apical microvilli and closely associated underlying lymphoid cells were observed in both RM and NALT (Figure 3C). Scanning electron microscopy further identified scattered RM epithelial cells with short, irregular microvilli. In the examined NALT sample, many surface epithelial cells lacked cilia and displayed prominent apical protrusions (Figure 3D). Together, these observations revealed cells at both antigen-sampling sites with morphological features similar to those of nasal M cells, as described in previous studies [18, 32, 33].

### RM and NALT show distinct OVA distribution kinetics

**Figure 4:**
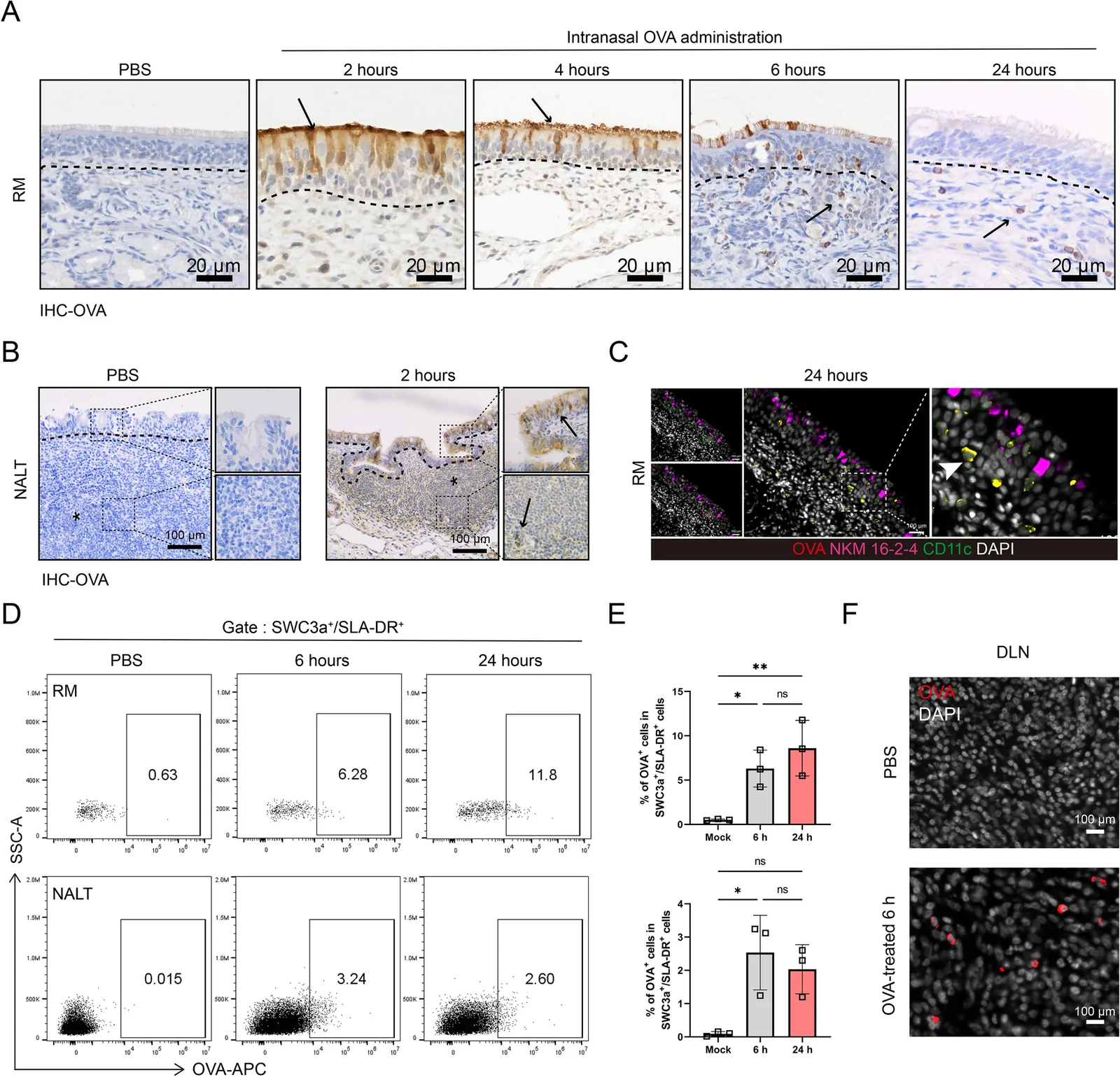
Spatiotemporal OVA distribution and uptake by putative APCs. A Ovalbumin (OVA) immunohistochemistry in respiratory mucosa (RM) at 2, 4, 6, and 24 h after intranasal administration to 7-day-old piglets. Arrows indicate OVA^+^ cells; dashed lines separate the epithelium (upper) from the lamina propria (lower). **B** Representative immunohistochemical images showing OVA^+^ cells in nasopharynx-associated lymphoid tissue (NALT). Dashed lines separate the epithelium (upper) from the lymphoid follicles (lower), and asterisks indicate lymphoid follicles. **C** Multiplex immunofluorescence showing OVA, NKM 16-2-4, and CD11c at 24 h. The boxed region is enlarged. **D** Representative flow cytometry plots of OVA^+^ cells within the SWC3a^+^/SLA-DR^+^ putative APC-enriched population isolated from RM and NALT at 6 and 24 h. PBS-treated piglets served as controls. **E** Quantification of OVA^+^ cells within the gated population. Data are mean ± SD (*n* = 3 piglets per group); one-way ANOVA followed by Tukey’s multiple-comparisons test. *P < 0.05; **P < 0.01; ns, not significant. **F** Representative immunofluorescence image showing OVA^+^ cells in the mandibular draining lymph nodes at 6 h after intranasal OVA administration.

To trace the spatiotemporal distribution of OVA in the porcine nasal mucosa, RM and NALT tissues were collected at 2, 4, 6, and 24 h after intranasal OVA administration. At 2 and 4 h, OVA was predominantly localized within the RM epithelium. By 6 h, OVA^+^ cells were also detected in the underlying lamina propria, whereas at 24 h, OVA signals were mainly observed in lamina propria cells (Figure 4A). In NALT, OVA was not only sampled by the follicle-associated epithelium (FAE; above the black dashed line) but was also detected within the underlying lymphoid follicle (below the black dashed line) as early as 2 h (Figure 4B). Nasal dendritic cells patrol the mucosa, processing and presenting foreign antigens [21, 34]. Consistent with this function, confocal microscopy revealed colocalization between OVA and CD11c signals in the nasal lamina propria at 24 h, which might include dendritic cells (Figure 4C). Furthermore, flow cytometric analysis identified OVA^+^ cells within a heterogeneous SWC3a^+^/SLA-DR^+^ putative APC-enriched population in both RM and NALT at 6 and 24 h (Figure 4D, Additional file 6B). In both tissues, the frequencies of OVA^+^ cells among the SWC3a^+^/SLA-DR^+^ population did not differ significantly between 6 and 24 h (Figure 4E). In addition, OVA^+^ cells were detected in the mandibular lymph nodes (Figure 4F). Taken together, these findings identify RM and NALT as two spatially distinct antigen-sampling sites with different temporal kinetics. Intranasally administered OVA was acquired by local immune cells within these regions and was also detected in the mandibular draining lymph nodes.

### Differentiated nasal epithelial cultures support OVA uptake and transepithelial transport

**Figure 5:**
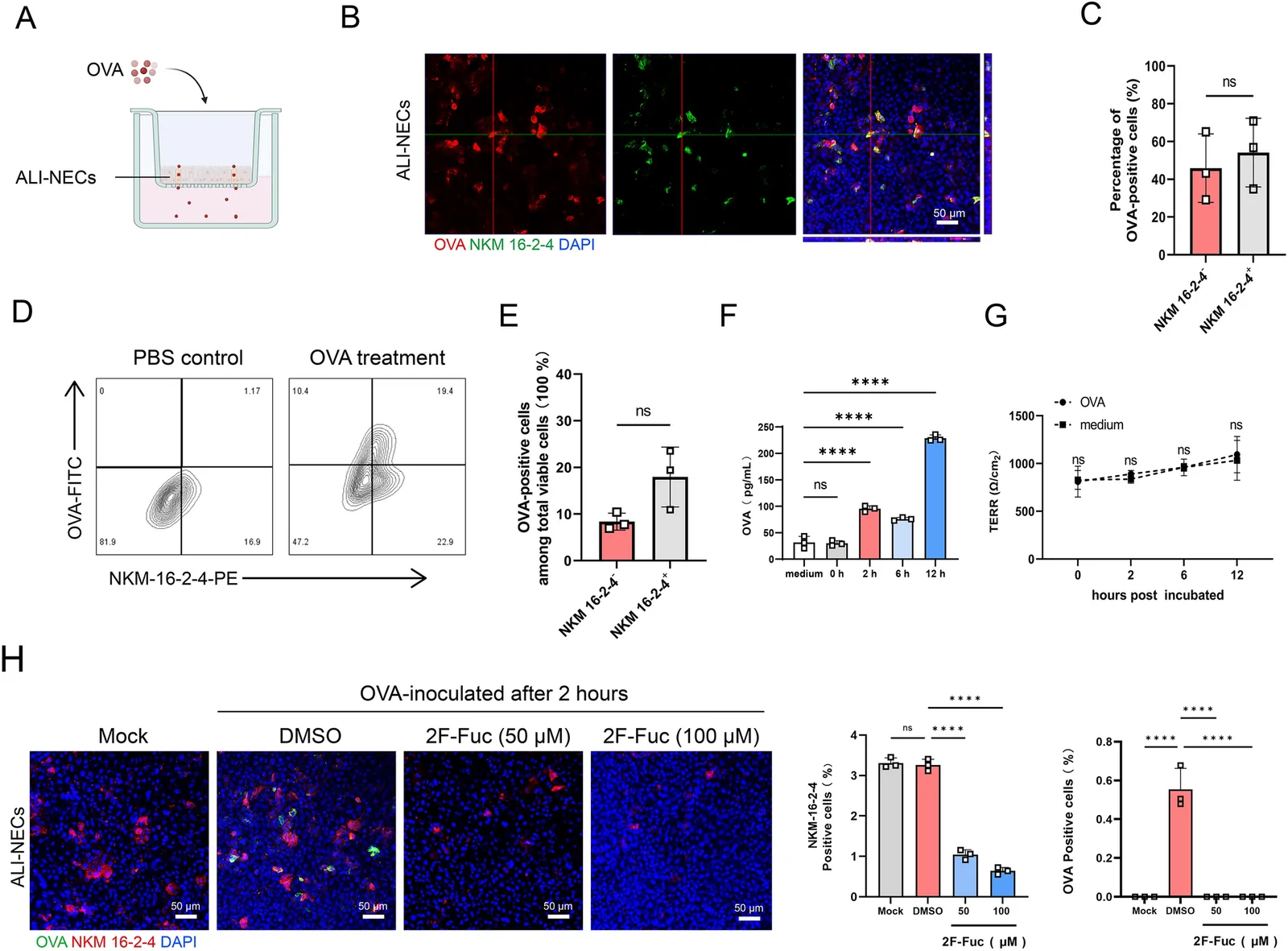
**Transepithelial OVA transport and fucosylation-associated uptake in ALI-NECs. A** Schematic of apical ovalbumin (OVA) exposure in differentiated air–liquid interface nasal epithelial cells (ALI-NECs). **B** Representative immunofluorescence images showing OVA and NKM 16-2-4 staining in ALI-NECs after 2 h of apical OVA exposure. **C** Image-based quantification of NKM 16-2-4^+^ and NKM 16-2-4^−^ cells among OVA^+^ epithelial cells. **D** Representative flow cytometry plots of OVA and NKM 16-2-4 staining in viable ALI-NECs. **E** Frequencies of OVA^+^/NKM 16-2-4^+^ and OVA^+^/NKM 16-2-4^−^ cells within viable ALI-NECs. **F** Basolateral OVA concentrations at the indicated times after apical exposure. **G** Transepithelial electrical resistance (TEER) changes during the experiment shown in (**F**). **H** OVA uptake and NKM 16-2-4 staining after 3 days of treatment with 50 or 100 μM 2F-Fuc. Quantification of NKM 16-2-4 signal and OVA^+^ cells is shown on the right. Dimethyl sulfoxide (DMSO) was the vehicle control. Data are presented as mean ± SD from three independent biological replicates, each derived from a different piglet. For panels (**C**) and (**E**), paired measurements were compared using two-tailed paired *t* tests. Data in panel (**F**) were analyzed using one-way repeated-measures ANOVA followed by Dunnett’s multiple-comparisons test, with the 0 h group as the reference. Data in panel (**G**) were analyzed using two-way repeated-measures ANOVA followed by Šídák’s multiple-comparisons test. Data in panel (**H**) were analyzed using one-way repeated-measures ANOVA followed by Dunnett’s multiple-comparisons test, with the DMSO (vehicle control)-treated group as the reference. ****P < 0.0001; ns, not significant.

To further characterize the functional properties of nasal M-like cells, we established an air–liquid interface culture model of primary porcine nasal epithelial cells (ALI-NECs). Differentiated ALI-NECs exhibited continuous junctional zonula occludens-1 (ZO-1) staining (Additional file 7A–D) and developed a pseudostratified columnar epithelium containing ciliated (Additional file 8) and goblet cells (Additional file 7E). In contrast, the swine tracheal epithelial cells (STEC) did not develop a pseudostratified epithelial architecture (Additional file 7D). α-(1, 2)-Fucosylation was readily detected in differentiated ALI-NECs but was not detectable in STEC (Additional file 7E). OVA signal frequently colocalized with α-(1,2)-fucosylated cells in ALI-NECs (Figure 5A, B), whereas no appreciable OVA uptake was detected in STEC (Additional file 7F). Notably, a subset of OVA-sampling epithelial cells lacked detectable α-(1, 2)-fucosylation (Figure 5C). Flow cytometric analysis showed that the frequency of OVA^+^/NKM 16-2-4^+^ cells was numerically higher than that of OVA^+^/NKM 16-2-4^−^ cells (17.93% versus 8.36%), although the difference was not statistically significant (Figure 5D, E).

To determine whether ALI-NECs could mediate transepithelial antigen transport, OVA was added to the apical chamber, and its concentration in the basolateral medium was measured at the indicated time points using separate Transwell inserts. Basolateral OVA concentrations increased over time and were highest at 12 h (Figure 5F). At each designated time point, TEER was measured in the corresponding Transwell inserts immediately before collection of the basolateral medium. TEER remained stable during the transport experiment (Figure 5G). To assess whether OVA transport involved a temperature-sensitive vesicular process, ALI-NECs were preincubated at 4 °C before OVA addition, as previously described [26, 35]. OVA transport across ALI-NECs was markedly reduced at 4 °C compared with that at 37 °C, supporting the involvement of a temperature-sensitive process consistent with vesicular/transcellular transport. By contrast, little or no basolateral OVA transport was detected across STEC monolayers (Additional file 7G). Together, the time-dependent appearance of OVA in the basolateral chamber, its reduction at 4 °C, the limited transport across STEC monolayers, and the maintenance of TEER support a contribution of temperature-sensitive vesicular transport without indicating overt disruption of epithelial barrier integrity. However, a contribution from paracellular transport cannot be excluded.

To investigate the association between cellular fucosylation and antigen sampling, ALI-NECs were treated with 2F-Fuc, a fucose analog that broadly inhibits cellular fucosylation and reduces the formation of fucosylated glycoconjugates. Cell Counting Kit-8 (CCK-8) assays and TEER measurements showed that treatment with up to 100 μM 2F-Fuc did not significantly affect cell viability or epithelial barrier integrity (Additional file 7H, I). Confocal microscopy showed that 2F-Fuc treatment significantly reduced OVA uptake by ALI-NECs, indicating that cellular fucosylation is associated with epithelial antigen sampling (Figure 5H).

### Fucosylation inhibition reduces OVA transfer to dBMCs

**Figure 6:**
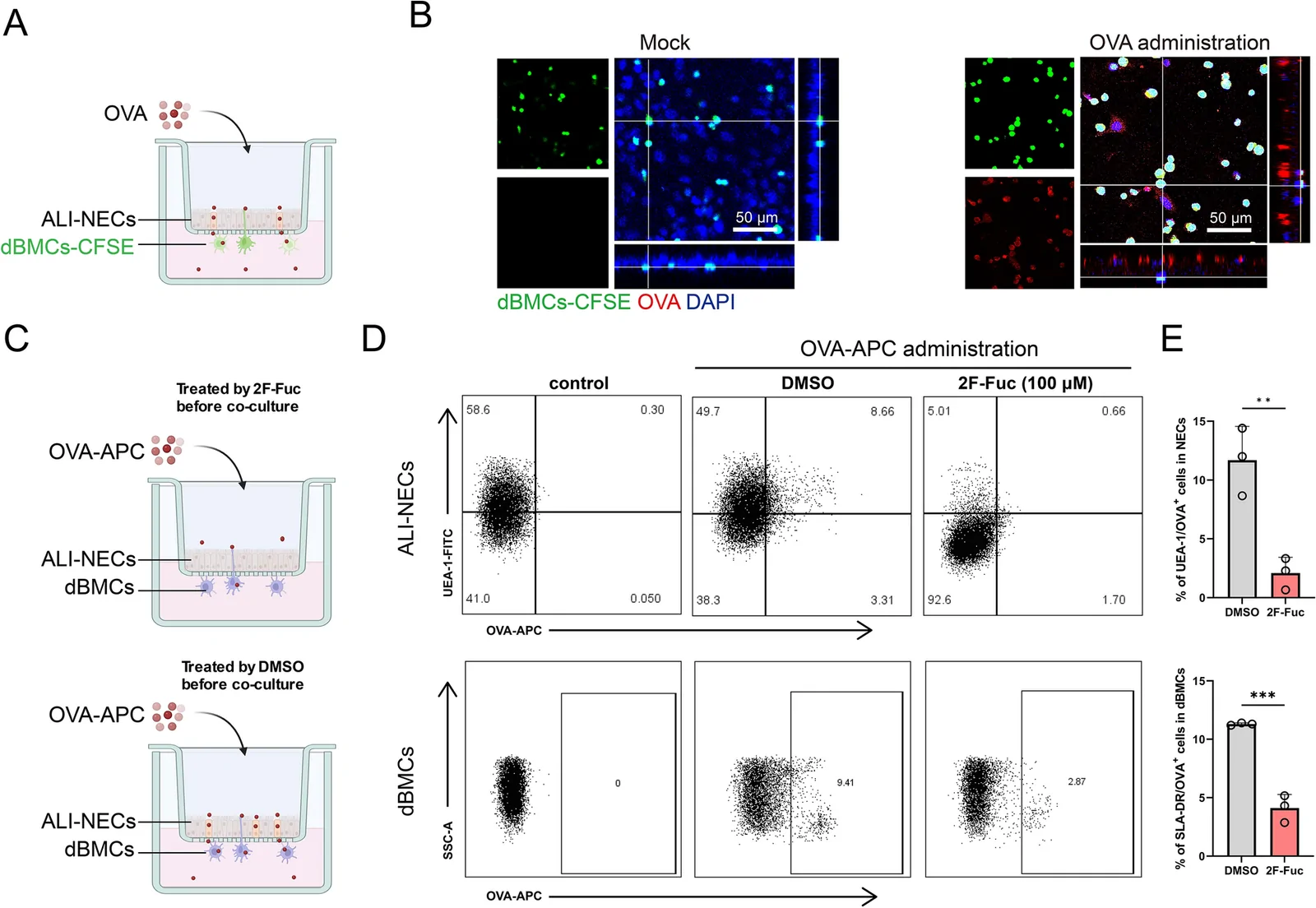
**Fucosylation inhibition reduces OVA transfer to dBMCs in co-culture. A** Schematic of the co-culture model. CFSE-labeled differentiated bone marrow cells (dBMCs) were seeded onto the basolateral side of the Transwell membrane beneath differentiated air–liquid interface nasal epithelial cells (ALI-NECs), followed by apical exposure to ovalbumin (OVA). **B** Orthogonal confocal views showing CFSE-labeled dBMCs beneath the ALI-NECs after apical exposure to OVA, with OVA^+^ dBMCs detected in the OVA-treated group. **C** Design of the 2F-Fuc inhibition experiment. ALI-NECs were treated with 100 μM 2F-Fuc or vehicle for 3 days before co-culture with dBMCs. OVA–APC (2 μg/mL) was added to the apical chamber for at least 2 h. After removal of OVA and washing, the cells were maintained under air–liquid interface conditions for an additional 6 h. Cells were harvested by trypsinization for flow cytometric analysis. **D** Representative flow cytometry plots of OVA uptake by UEA-1^+^ NECs or dBMCs (pregated on SWC3a^+^/SLA-DR^+^ cells). DMSO was used as the vehicle control. **E** Quantification of the populations shown in (**D**). Data are mean ± SD; *n* = 3 independent piglet-derived cultures; two-tailed paired *t* test. **P < 0.01; ***P < 0.001.

To assess epithelial-to-immune-cell antigen transfer, ALI-NECs were co-cultured with IL-4/GM-CSF-differentiated bone marrow cells (dBMCs), a heterogeneous population enriched for cells with antigen-presenting features (Figure 6A). After apical OVA exposure, OVA^+^ CFSE-labeled dBMCs were observed beneath the epithelial layer by confocal microscopy (Figure 6B). In the fucosylation–inhibition experiment (Figure 6C), 2F-Fuc pretreatment reduced the frequency of OVA^+^ UEA-1^+^ NECs and decreased OVA acquisition by swine leukocyte antigen DR (SLA-DR)^+^ dBMCs (Figure 6D, E). These findings indicate that the differentiated nasal epithelium can facilitate antigen transfer to underlying dBMCs and that this process is sensitive to inhibition of cellular fucosylation.

## Discussion

**Figure 7:**
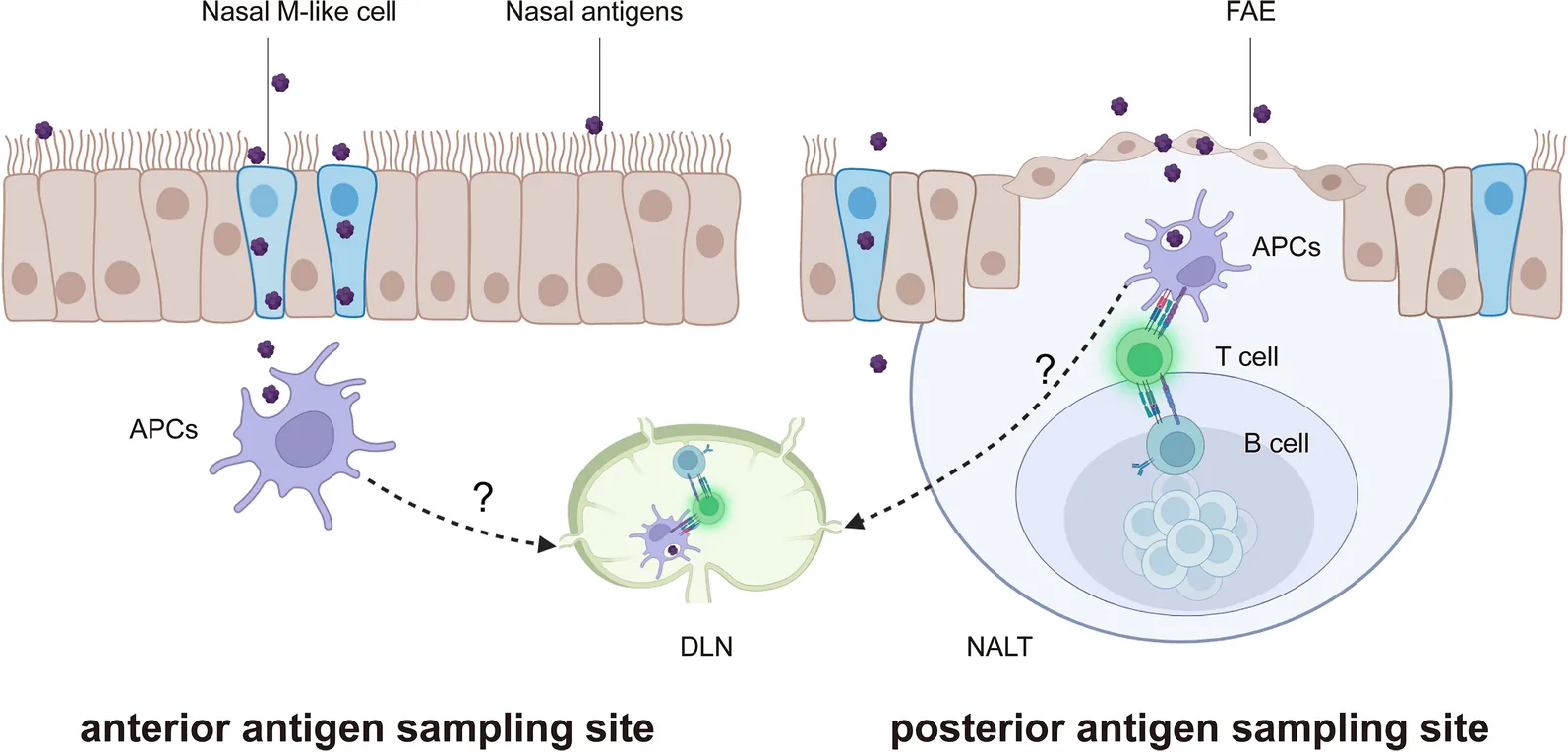
**Schematic of two antigen-sampling sites in the porcine nasal cavity.** Created with BioRender.com (agreement number: BU2A9HBZ01). The anterior respiratory mucosa (RM) and posterior nasopharynx-associated lymphoid tissue (NALT) form complementary antigen-sampling compartments. In anterior RM, scattered epithelial cells with M-cell-like features transport luminal protein antigen toward underlying immune cells. In posterior NALT, antigens cross the follicle-associated epithelium and rapidly reach the organized lymphoid follicle. Antigen or antigen-bearing cells might subsequently reach mandibular draining lymph nodes.

Antigen sampling is a critical early event in mucosal responses to intranasally delivered protein antigens [36]. In this study, we identified the anterior respiratory mucosa (RM) and posterior nasopharynx-associated lymphoid tissue (NALT) as two spatially distinct antigen-sampling sites with different transport kinetics: rapid delivery to the NALT lymphoid follicle and slower movement from the RM epithelium to the lamina propria. In vivo, OVA-sampling epithelial cells at both sites exhibited M-cell-like features, and a similar antigen-sampling population was recapitulated in differentiated ALI-NECs in vitro. The ALI-NECs model further supported transepithelial OVA transport and antigen transfer to underlying dBMCs, both of which were associated with cellular fucosylation. Together, these findings reveal two spatially distinct antigen-sampling compartments for early nasal antigen sampling and transfer in pigs (Figure 7).

RM is covered by a pseudostratified ciliated columnar epithelium that restricts macromolecular penetration [24, 37]. Nevertheless, nasal M-like cells are sparsely distributed throughout RM and might provide specialized portals for delivering luminal antigens to antigen-presenting cells within the underlying lamina propria. Ultrastructural analyses using transmission and scanning electron microscopy revealed epithelial cells with apical membrane invaginations and irregular microvilli in the porcine nasal cavity, resembling the morphology of nasal M cells previously described in mice [16, 18]. Although α-(1, 2)-fucosylation has been used to identify subsets of both rodent and porcine nasal M-like cells, this glycan feature could not be directly integrated with scRNA-seq data. Moreover, reanalysis using M-cell-associated genes identified in rodents and humans failed to resolve a discrete porcine-M-cell subset. This discrepancy might reflect species- and tissue-specific differences in M cell transcriptional programs, as well as the limited specificity of currently available markers. Thus, additional studies are required to identify more specific markers and define the transcriptional identity of porcine nasal M-like cells.

OVA reached the NALT lymphoid follicle by 2 h, whereas in RM it remained predominantly epithelial at 2–4 h and was detected in the lamina propria from 6 h onward. These differences likely reflect regional epithelial architecture and immune-cell organization. The epithelium overlying NALT is positioned directly above a densely cellular lymphoid compartment, facilitating rapid access of sampled antigen. In contrast, the thicker pseudostratified RM and its more dispersed immune-cell population might delay epithelial-to-lamina-propria transfer [38]. Nevertheless, the broad distribution of scattered antigen-sampling cells in RM might provide an additional route for uptake of intranasally delivered proteins outside organized NALT [37, 39]. Consistent with this possibility, the NEC–dBMC co-culture model demonstrated that antigens sampled by the epithelium could subsequently be acquired by underlying immune cells. However, this simplified system does not fully reproduce the cellular and extracellular microenvironment of the nasal mucosa, and the duration of co-culture was limited by the absence of matrix or stromal support. Previous studies have shown that mucosal dendritic cells can migrate to draining lymphoid tissues after antigen uptake, thereby linking peripheral antigen acquisition to adaptive immune activation [39]. OVA^+^ cells were detected in mandibular-draining lymph nodes, but the present experiments do not establish whether OVA was transported by migratory nasal dendritic cells, by other leukocytes, or in cell-free form. Fate mapping, selective cell labeling, or photoconvertible antigen-tracking approaches will be required to define the cellular route from nasal mucosa to draining lymph nodes.

In vitro, a population of α-(1, 2)-fucosylation^+^ nasal M-like cells was identified in differentiated ALI-NECs. Pharmacological inhibition of fucosylation reduced OVA uptake by ALI-NECs without compromising cell viability or epithelial barrier integrity, suggesting that cellular fucosylation contributes to nasal epithelial antigen sampling. However, because 2F-Fuc broadly suppresses cellular fucosylation rather than selectively targeting α-(1, 2)-linked fucose residues, these findings do not establish a direct causal role for α-(1, 2)-fucosylation in M-like cell-mediated antigen uptake [40]. Notably, a substantial fraction of OVA^+^ cells lacked detectable α-(1, 2)-fucosylation, indicating that antigen sampling is not restricted to this phenotype. Alternatively, α-(1, 2)-fucosylation might be dynamically regulated during M-like cell differentiation or following antigen exposure, resulting in heterogeneous or temporally variable expression. Therefore, although α-(1, 2)-fucosylation might help to identify or enrich for a subset of porcine nasal M-like cells, it appears to have insufficient specificity to serve as a standalone marker for the entire antigen-sampling population. The biological function of α-(1, 2)-fucosylation in nasal antigen sampling also remains unclear. It might influence the expression, localization, or activity of surface glycoproteins involved in antigen binding, endocytosis, vesicular trafficking, or transcytosis. Future studies should therefore combine α-(1, 2)-fucosylation with additional phenotypic and functional markers and employ more selective genetic or enzymatic approaches to determine its specific contribution to antigen uptake.

In summary, our findings identify the anterior RM and posterior NALT as two spatially distinct antigen-sampling sites in the porcine nasal cavity (Figure 7). NALT enabled rapid antigen access to the underlying lymphoid follicle, whereas antigen transfer across RM occurred more gradually and involved sparsely distributed nasal M-like cells and underlying antigen-presenting cells. Although α-(1, 2)-fucosylation was associated with antigen uptake, it was not sufficiently specific to identify the entire antigen-sampling population. These findings reveal a dual-site architecture of porcine nasal antigen sampling that may support the early stages of mucosal immune induction and provide a basis for improving intranasal vaccine delivery in pigs.

## Supplementary information

**Additional File 1:** ** Overview of pigs and porcine samples used in this study.**

**Additional File 2: Antibody and lectin used in this study.**

**Additional File 3:** **Marker genes used in scRNA-seq analysis.**

**Additional File 4:** **NKM 16-2-4+ cells in the porcine respiratory tract**. **A** Upper respiratory tract including the nasal respiratory mucosa (RM), olfactory mucosa (OM), trachea mucosa, and lung tissue from 7-day-old piglets were stained by NKM 16-2-4 (green). Cell nuclei were stained with DAPI (blue). **B** IFA was performed to investigate NKM 16-2-4^+^ cells in piglets of different ages (0, 10, 30, 60, and 180-day-old). Cell nuclei were stained with DAPI (blue).

**Additional File 5:** **Single-cell atlas of the porcine nasal mucosa.** **A** *t*-Stochastic neighbor embedding (tSNE) plots displaying 15 455 cells from porcine nasal mucosa identified as 17 distinct cell types. **B** Dot plots showing marker genes for nasal cell types, with the fraction of expressing cells and average expression within each cell type indicated by dot size and color, respectively. **C** Dot plots showing marker genes for M cells, with the fraction of expressing cells and average expression within each cell type.

**Additional File 6:** **Representative flow cytometric gating strategies used for the analyses presented in the main figures.** **A** Gating strategy for nasal swab-derived cells used for the flow cytometric analysis shown in Figure 2D. Cells were first gated according to their forward scatter (FSC-A) and side scatter (SSC-A) characteristics to exclude debris, followed by the selection of singlets (FSC-H and FSC-A)and viable cells (Zombie NIR). **B** Gating strategy for cells isolated from the respiratory mucosa (RM) and nasopharynx-associated lymphoid tissue (NALT), corresponding to the analysis shown in Figure 4D. After the exclusion of debris, doublets, and dead cells, SWC3a^+^/SLA-DR^+^ cells were identified as a putative myeloid antigen-presenting cell-enriched population. 7-AAD was used to exclude the dead cells. **C** Gating strategy for in vitro air–liquid interface nasal epithelial cells (ALI-NECs) used for the analysis shown in Figure 5D. NECs were sequentially gated on the basis of scatter characteristics, singlet discrimination, and cell viability (7-AAD). **D** Gating strategy for NECs (upper) and differentiated bone marrow cells (dBMCs; lower) used for the analysis shown in Figure 6D. Following the exclusion of debris, doublets, and dead cells (7-AAD), SWC3a^+^/SLA-DR^+^ cells were identified for subsequent analysis. Numbers within or adjacent to the gates indicate the percentage of cells relative to the corresponding parent population.

**Additional File 7:** **Characteristics of porcine ALI-NECs**. **A** Representative images showed the porcine primary nasal epithelial cells (NECs) cultures observed by light microscopy in liquid–liquid or air–liquid conditions. **B** NECs were initially cultured in expansion medium under liquid–liquid conditions and transepithelial electrical resistance (TEER) was monitored daily. Once TEER stabilized, the medium was switched to differentiation medium. Subsequently, the apical medium was removed to establish air–liquid interface (ALI) conditions. **C** Immunofluorescence images showed that ALI-NECs expressed the tight and tight junction protein ZO-1 (green). **D** H&E staining showing the representative image of swine tracheal epithelial cells (STEC) line and ALI-NECs after differentiation, as well as porcine nasal tissue. **E** Immunofluorescence images showed cilia (α-tubulin; green) and goblet cell (MUC5AC; green) in ALI-NECs. **F** immunofluorescence staining showed the OVA and NKM 16-2-4 distribution in STEC after adding OVA for 2 h. **G** Transwell inserts containing ALI-NECs or STEC were preincubated at 4 °C or 37 °C for 30 min before OVA addition and maintained at the respective temperatures throughout the transport assay. The lower-chamber medium was collected 2 h after OVA addition, and the OVA concentration was measured by ELISA. Mean ± SD (*n* = 3); one-way ANOVA. **H** Viability of ALI-NECs treated with 2F-Fuc using the CCK-8 assay. Mean ± SD (*n* = 3); one-way ANOVA followed by Dunnett’s multiple-comparisons test. **I** TEER change of ALI-NECs after 2F-Fuc Treatment. Mean ± SD (*n* = 3); two-tailed unpaired *t* tests. ***P < 0.001; **P < 0.01; *P < 0.05; ns, not significant.

**Additional File 8:** **Movie of ciliated movement in ALI-NECs.**

## Data Availability

All data generated or analyzed during this study are included in the published paper and its supplementary information files.
