# Supplementary material for: Dual antigen-sampling sites support early mucosal antigen uptake in the porcine nasal cavity
Source: Vet Res. 2026 Oct 27;57:187. doi: 10.1186/s13567-026-01863-2 (PMC13715195; doi:10.1186/s13567-026-01863-2)
Supplement: Supplementary file 1 [file 13567_2026_1863_MOESM1_ESM.docx]

| **Experimental purpose** | **Age and number** | **Sex** | **Treatment and sampling** | **Samples collected** | **Methods and related figures** |
| --- | --- | --- | --- | --- | --- |
| Identification of OVA-uptake sites and phenotypic characterization of OVA-positive cells in the nasal mucosa | Six 7-day-old piglets (*n* = 3/group) | Sex was not recorded | Intranasal OVA or PBS administration; sampled 2 h later | RM and NALT | IHC and IF; Figures 1A, 2A, and 2B |
| Assessment of the distribution of OVA-positive cells across different regions of the nasal cavity | Three 7-day-old piglets (*n* = 3) | Sex was not recorded | Intranasal OVA administration; sampled 2 h later | Longitudinal nasal tissue blocks | H&E staining and IF; Figure 1B |
| Assessment of the age-related distribution of OVA-positive cells in the nasal mucosa | Newborn and 7-, 10-, and 30-day-old piglets (*n* = 2 per age) | Sex was not recorded | Intranasal OVA administration; sampled 2 h later | RM from region III of the nasal cavity | IF; Figure 1D |
| Flow cytometric characterization of OVA-positive nasal swab-derived cells | Six 7-day-old piglets (*n* = 3/group) | Sex was not recorded | Intranasal OVA or PBS administration; sampled 2 h later | Nasal epithelial cells collected by nasal swabbing | Flow cytometry; Figure 2D |
| Ultrastructural characterization of the porcine nasal mucosa | One healthy 7-day-old piglet | Sex was not recorded | No experimental treatment | RM and NALT | TEM and SEM; Figure 3C and D |
| Time-course analysis of OVA distribution in RM, NALT, and DLN | Fifteen 7-day-old piglets (*n* = 3/group) | Sex was not recorded | Intranasal OVA administration; sampled at 2, 4, 6, or 24 h. PBS-administered piglets served as controls | RM and NALT at the indicated time points; draining lymph nodes (DLNs) at 6 h | IHC and IF; Figures 4A-C and 4F |
| NKM 16-2-4 positive cells in respiratory tracts and RM from different ages of pigs | Newborn and 10-, 30-, 60-, and 180-day-old pigs (*n* = 2) | Sex was not recorded | No experimental treatment | RM, OM, NALT, trachea, and lung | IF; Supplemental Figure S1 |
| Isolation of bone marrow cells and nasal epithelial cells | Three healthy 30-day-old piglets | Sex was not recorded | No experimental treatment | Nasal mucosa of turbinate and femurs | Corresponding Materials and Methods sections |

**Supplemental table 1. Overview of pigs and porcine samples used in this study**

Abbreviations: OVA, ovalbumin; PBS, phosphate-buffered saline; RM, respiratory mucosa; NALT, nasopharynx-associated lymphoid tissue; DLN, draining lymph node; IHC, immunohistochemistry; IF, immunofluorescence; H&E, hematoxylin and eosin; TEM, transmission electron microscopy; SEM, scanning electron microscopy; nasal olfactory mucosa (OM).
