# Supplementary material for: Dual antigen-sampling sites support early mucosal antigen uptake in the porcine nasal cavity
Source: Vet Res. 2026 Oct 27;57:187. doi: 10.1186/s13567-026-01863-2 (PMC13715195; doi:10.1186/s13567-026-01863-2)
Supplement: Supplementary file 2 [file 13567_2026_1863_MOESM2_ESM.docx]

**Additional file 2. Antibody and lectin used in this study**

| **Antibody/reagent** | **Clonality/catalog** | **Host species** | **Reported Species reactivity** | **Basis for porcine use/validation** | **Application** | **Conjugate** | **Dilution** | **Supplier** |
| --- | --- | --- | --- | --- | --- | --- | --- | --- |
| Anti-OVA antibody | polyclonal/NB600-922 | rabbit | chicken | Used to detect exogenously administered chicken OVA in porcine tissues; PBS-treated and/or no-primary-antibody controls showed no specific signal. | IHC/IF | none | 1:200 | NOVUS, USA |
| Anti-OVA antibody | monoclonal/ab306591 | rabbit | chicken | Used to detect exogenously administered chicken OVA in porcine tissues; PBS-treated and/or no-primary-antibody controls showed no specific signal. | Flow (Intra) | none | 1:100 | Abcam, UK |
| Anti-KRT5 antibody | polyclonal/A2662 | mouse | human | Not formally validated for pig; staining was evaluated based on the expected basal epithelial localization. | IF | none | 1:200 | ABclonal, China |
| Anti-MUC5AC antibody | monoclonal/852F7F6 | mouse | human | Not formally validated for pig; staining was evaluated based on the expected distribution and morphology of goblet cells. | IF | none | 1:200 | Abcepta, China |
| Anti-OMP antibody | monoclonal/EPR19190 | mouse | human | Not formally validated for pig; staining was evaluated based on the characteristic morphology and localization of olfactory sensory neurons. | IF | none | 1:200 | Abcam, UK |
| Anti-α-Tubulin antibody | monoclonal/66200-1-Ig | mouse | pig | Manufacturer-reported reactivity with pig; expected apical ciliary staining was observed. | IF | none | 1:200 | Proteintech, China |
| NKM 16-2-4 antibody | monoclonal/130-096148/  130-102-150 | rat | mouse | Reported for mouse; used to detect α-(1,2)-fucosylated glycans in porcine tissues, but not validated as a porcine M-cell-specific marker. | IF/Flow | none/PE | 1:20/1:10 | Miltenyi Biotec, Germany |
| UEA-1 lectin | lectin/L9006 | *ulex europaeus* | / | Not applicable; a lectin recognizing α-(1,2)-fucosylated glycans rather than a species-specific protein antigen. | IF/Flow | FITC | 1:500 | Sigma, Germany |
| Anti-CD11c antibody | monoclonal/AFKL0075 | rabbit | mouse | Not formally validated for pig; staining was interpreted cautiously based on the distribution and morphology of CD11c-positive cells. | IF | none | 1:200 | Aifang, China |
| Anti-SLA-DR antibody | monoclonal/154296 | mouse | pig | Manufacturer-reported reactivity with pig. | Flow | FITC | 1:10 | BIO-RAD, USA |
| Anti-SWC3a antibody | monoclonal/74-22-15A | mouse | pig | Manufacturer-reported reactivity with pig. | Flow | PE | 1:100 | BD, USA |
| OVA-FITC | bs-0283P-FITC | / | / | / | Flow | FITC | 2 μg/mL | BIOSS, China |
| OVA-APC | bs-0283P-APC | / | / | / | Flow | APC | 2 μg/mL | BIOSS, China |
| Anti-rabbit IgG-YSFluor® 594 | polyclonal/33712ES60 | Goat | rabbit | / | IF | YSFluor® 594 | 1:200 | YESEN, China |
| Anti-rabbit IgG-FITC | polyclonal/33707ES60 | Goat | rabbit | / | IF | FITC | 1:200 | YESEN, China |
| Anti-rabbit IgG-APC | polyclonal/771445ES60 | Goat | rabbit | / | Flow | APC | 1:200 | YESEN, China |
| Anti-Rat IgG-YSFluor® 647 | polyclonal/34413ES60 | Donkey | rat | / | IF | YSFluor® 647 | 1:200 | YESEN, China |
| Anti-mouse IgG-YSFluor® 488 | polyclonal/33206ES60 | Donkey | mouse | / | IF | YSFluor® 488 | 1:200 | YESEN, China |
