# Supplementary material for: Dual antigen-sampling sites support early mucosal antigen uptake in the porcine nasal cavity
Source: Vet Res. 2026 Oct 27;57:187. doi: 10.1186/s13567-026-01863-2 (PMC13715195; doi:10.1186/s13567-026-01863-2)
Supplement: Supplementary file 3 [file 13567_2026_1863_MOESM3_ESM.docx]

**Additional file 3. Marker genes used in scRNA-seq analysis**

| Cell type | Marker gene | Reference |
| --- | --- | --- |
| Basal | KRT5, TP63, DLK2 | [27] |
| Goblet | SCGB1A1, MUC5AC | [27] |
| Cilia | FOXJ1, TPPP3 | [27] |
| Serous | LTF, PEBP4 | [27] |
| Duct | ALDH1A3, SOX9 | [27] |
| Fibroblasts | COL1A1 | [27] |
| Chondrocytes | COL1A1, ACAN, MMP13 | [27] |
| Endothelial | VWF, PECAM1, CDH5 | [27] |
| Granulocyte | CSF3R, CXCR2, S100A9 | [27] |
| Smooth muscle | CALD1, DES | [27] |
| Dendritic/macrophage | CD80, CD86, CD163 | [27] |
| Natural killer | NKG7, SAMD3, GNLY | [27] |
| T cell | CD3D, CD3E | [27] |
| Ionocyte | CFTR, FOXI1 | [27] |
| Club lineage | CDHR3, FOXJ1, MUC5AC | [27] |
| Suprabasal | KRT4, low expression of KRT5 and TP63 | [27] |
| Microfold cell^*^ | SOX8, SPIB, CCL9, CCL20, TNFRSF11A, TNFAIP2, MARCKSL1, ANXA5, RELA, RELB, GP2, KRT18 | [29], [30],[31] |

“*”: microfold cell associated genes previously reported in human and mouse studies.
